# Chronic Lung Allograft Dysfunction Post Lung Transplantation: A Review of Computed Tomography Quantitative Methods for Detection and Follow-Up

**DOI:** 10.3390/jcm10081608

**Published:** 2021-04-10

**Authors:** Trieu-Nghi Hoang-Thi, Guillaume Chassagnon, Thong Hua-Huy, Veronique Boussaud, Anh-Tuan Dinh-Xuan, Marie-Pierre Revel

**Affiliations:** 1 AP-HP.Centre, Hôpital Cochin, Department of Radiology, Université de Paris, 75014 Paris, France; htrieunghi@yahoo.fr (T.-N.H.-T.); guillaume.chassagnon@aphp.fr (G.C.); 2 Department of Diagnostic Imaging, Vinmec Central Park Hospital, Ho Chi Minh City 70000, Vietnam; 3 AP-HP.Centre, Hôpital Cochin, Department of Respiratory Physiology, Université de Paris, 75014 Paris, France; huy-thong.hua@aphp.fr (T.H.-H.); anh-tuan.dinh-xuan@aphp.fr (A.-T.D.-X.); 4 AP-HP.Centre, Hôpital Cochin, Department of Pneumology, Université de Paris, 75014 Paris, France; veronique.boussaud@aphp.fr

**Keywords:** lung transplantation, image processing, computer-assisted, pulmonary disease, chronic obstructive, bronchiolitis obliterans

## Abstract

Chronic lung allograft dysfunction (CLAD) remains the leading cause of morbidity and mortality after lung transplantation. The term encompasses both obstructive and restrictive phenotypes, as well as mixed and undefined phenotypes. Imaging, in addition to pulmonary function tests, plays a major role in identifying the CLAD phenotype and is essential for follow-up after lung transplantation. Quantitative imaging allows for the performing of reader-independent precise evaluation of CT examinations. In this review article, we will discuss the role of quantitative imaging methods for evaluating the airways and the lung parenchyma on computed tomography (CT) images, for an early identification of CLAD and for prognostic estimation. We will also discuss their limits and the need for novel approaches to predict, understand, and identify CLAD in its early stages.

## 1. Introduction

Compared to other organ transplantation, lung transplantation remains associated with a poorer prognosis, with a median survival of only six years after transplantation [1]. The main cause of death beyond the first year is chronic lung allograft dysfunction (CLAD). This term refers to the deterioration of lung function as a result of chronic rejection and is not limited to an exclusively obstructive phenotype, referred to as bronchiolitis obstructive syndrome (BOS), but also includes a restrictive phenotype of chronic allograft dysfunction, termed restrictive allograft syndrome (RAS). The classification of CLAD phenotypes updated in 2019 by the International Society of Heart and Lung Transplantation (ISHLT) included BOS, accounting for 70% of allograft dysfunctions, and three other entities, RAS, mixed, and undefined phenotypes, representing the other 30% of phenotypes [2]. Their respective definitions are summarized in Table 1.

Hota et al. provided a comprehensive description of the different CLAD phenotypes on high-resolution CT [3]. RAS is associated with persistent parenchymal opacities and pleural thickening showing upper lung predominance, with pleuro parenchymal fibroelastosis on pathological examination, explaining the restrictive pattern [2,3]. Complementing pulmonary function tests (PFTs) with CT is thus mandatory not only to exclude other causes of lung function degradation but also to classify CLAD phenotypes. This classification is important for prognosis, with patients with a restrictive pattern of CLAD having worse survival [4]. Furthermore, Levy et al. [5] demonstrated that patients with undefined/unclassified CLAD phenotypes who had RAS-like opacities on chest imaging had significantly worse allograft survival than patients with the BOS phenotype. Suhling et al. [6] performed visual scoring of reticulations and consolidations in lung transplanted (LTx) patients and found poorer survival in patients with severe reticular changes. Imaging follow-up is especially important to detect CLAD in patients with single lung transplantation, because native lung disease may confound the interpretation of physiology [7]. 

**Figure 1:**
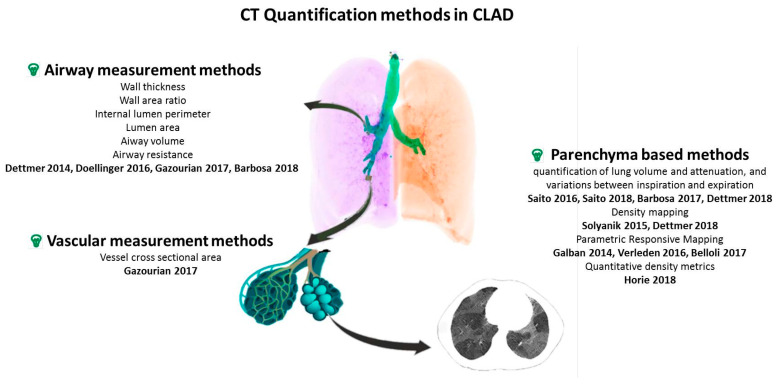
Overview of CT quantification methods for CLAD. CT: computer tomography, CLAD: chronic lung allograft dysfunction. Airway measurement methods [8,9,12,14], Vascular measurement methods [14], Parenchyma based methods [10,11,15,16,17,18,19,20,21].

A switch from visual descriptive evaluation to quantitative automatic measurements has recently emerged in the field of medical imaging. Quantitative imaging has been mainly used for the evaluation of BOS, the obstructive phenotype of CLAD. The implementation of such imaging analysis tools for CLAD evaluation in LTx patients either directly confirms airway thickening and vascular remodeling in the allograft lung [8,9] or indirectly suggests their occurrence through air-trapping quantification [10,11]. Applying machine learning to imaging data analysis may further improve the management of CLAD [12]. In this review article, we will provide an overview of the CT quantification methods used to investigate CLAD of various phenotypes (see Figure 1 and Table 2), and discuss novel approaches to identify CLAD in its early stage. As sub-clinical chronic rejection compromises allograft integrity, there is a need to develop physiological and imaging tests allowing early detection of CLAD, with a potential role for quantitative imaging to serve as a biomarker, allowing early identification of CLAD [13].

## 2. Airway Measurement Methods: Computer-Assisted Airway Morphometry

Since the most common phenotype of CLAD is that of BOS, quantitative methods allowing the direct assessment of the airways have been developed. BOS primarily affects the small airways, with diameters of less than 2 mm. Due to the limited spatial resolution of clinical CT scanners, new measurement methods had to be developed to allow the measurement of the density and wall thickness of the small airways [22]. Such a method made it possible to obtain values of the wall thickness of the small airways correlated with FEV1 in COPD patients [23]. Several software now available commercially make it possible to obtain an automatic segmentation of the bronchial tree and sections strictly perpendicular to the bronchial axis considered at different levels, as well as an automatic measurement of the bronchial wall thickness (WT) and the wall area ratio (WA%), the ratio of the airway wall area to the whole airway area. 

Using the YACTA software [24], Doellinger et al. retrospectively examined a total of 2190 airway cross sections on CT scans performed within 11 years of follow-up of 26 LTx patients with at least one measurement in every lobe of the transplanted lung [8]. They showed significant differences between patients with and without BOS in term of changes over time of WA% and WT and concluded that bronchial wall thickening and luminal dilatation observed in lung transplant allograft rejection can be detected and quantified using computer-assisted airway morphometry. Gazourian et al., in a retrospective analysis of 22 patients, observed and increase in the air airway internal lumen perimeter (PI), and airway lumen area (AI) in the 13 patients developing BOS [14]. They used Airway Inspector (now renamed Chest Imaging Platform) for evaluating the third and fourth generation B1 and B10 airways. They also evaluated the pulmonary vessels cross-sectional areas (CSA) after upper thresholding (cut-off level of −500 HU) and use of a connected components technique on volumetric CT pulmonary angiogram to isolate the vessel cross sections. These authors also measured the airway/vessel ratios (A/V ratio) on non-volumetric CT scans. They found a statistically significant decrease in the vessel CSA in BOS patients and a significant increase in the airway/vessel ratio in BOS progressors. This observation is in line with known physiology, namely vasoconstriction in poorly ventilated areas due to small airways disease. These two studies (Doellinger [8] and Gazourian [14]), although both based on a limited number, showed differences between BOS and controls, but automated quantification of airway dimension was limited by the need for manual adjustments. In addition, the methods used do not translate into early identification or prognostic tools for CLAD. 

Dettmer [9] evaluated 25 patients with BOS and 116 controls using MeVis Airway Examiner. WA% on inspiration was significantly greater in patients with BOS, but the variability of bronchial wall measurements was high and the values for the WA% on inspiration in patients with and without BOS overlapped considerably, due to variable underlying lung volumes. Even though this limitation could be overcome by performing spirometry-controlled CT acquisitions, the authors concluded to a limited value of WA% for establishing a diagnosis of BOS in individual patients. 

Barbosa et al. [12] conducted a retrospective analysis of LTx patients who had paired inspiratory and expiratory CTs, with an objective to detect BOS 0-p stage, as indicator of early disease (≥10% decline in FEV1 or ≥25% decline in forced expiratory flow 25–75%). Baseline prediction of BOS was performed in a cohort of 41 patients, of which 15 developed BOS. They measured the airway volumes and airway resistances after 3D analysis of the airways and lung lobes using three different commercially available software, Mimics, TGrid 14.0 and Fluent 14.0, for computational fluid dynamics simulations. The authors found that BOS patients experienced an increase in central airway volume on expiratory CT, whereas patients with other cause of FEV1 decline had a decrease in the central airway volume on inspiratory CT. According to the authors, these findings reflected an increase in the extent of air trapping and peribronchiolar fibrosis which characterizes the BOS phenotype of CLAD. Image post-processing included segmentation of the tracheobronchial tree down to the level of airways with a diameter of 1–2 mm which required manual correction and took 2 to 6 h per scan, which is the main limitation to consider the clinical use of this approach.

## 3. Lung Parenchyma Methods: Assessment of Lung Volume and Attenuation, and Their Variations between Inspiration and Expiration

Barbosa et al. [16], using the Advanced Normalization Tools (ANTs) software package, calculated the aerated lung volume in inspiration and expiration via exclusion of any voxel >−50 HU and then evaluated the volume change between inspiration and expiration as the difference between these two values, in a retrospective patient cohort excluding the RAS phenotype. In addition to volume change, two other quantitative parameters reflecting air trapping were evaluated: the volume of voxels with attenuation <−856 HU on expiration and the volume of voxels with an increase in attenuation <75 HU from inspiration to expiration following non-rigid registration between the inspiratory and expiratory imaging datasets. Only 59% of quantitative CT metrics were significantly correlated with BOS status in this study including 176 LTx patients, and none of the variables alone was a good predictor of BOS. However, a support vector machine (SVM) model based on the quantitative CT variables outperformed models using visual scoring of CT anomalies or PFT for BOS prediction after unilateral LTx.

Dettmer et al. [17] conducted a prospective evaluation with the objective to detect the BOS phenotype of CLAD in a cohort of 122 LTx patients. They performed CT acquisition with a spirometry-controlled technique at full inspiration and end of expiration and used Mevis Pulmo software, allowing volume measurement and histogram analysis (mean lung density/MLD, peak, percentiles) for the whole lung and separately for each lobe. They demonstrated that in patients with early-stage BOS, the lower lobe volume increased and the MLD decreased between the baseline and follow-up examinations, whereas the volume and MLD in the upper lobes remained nearly constant. The histogram parameters showing the highest accuracies for early BOS detection were the 10th percentile on expiration (AUC: 0.903) and the expiratory-to-inspiratory (E/I) MLD ratio (AUC: 0.886).

E/I MLD ratio is one of the multiple quantitative parameters used to evaluate the volume of air trapping in BOS. Solyanik et al. [15] evaluated two other automated methods to quantify air trapping, which had only slight differences with those previously mentioned in the study by Barbosa [16]: the lung volume having attenuation values ranging from −950 to −790 HU on expiratory CT scans (attenuation < −856 HU for Barbosa) and the lung volume with less than 80 HU change (<75 HU change for Barbosa) between inspiration and expiration. The latter requires non-rigid registration of inspiration-expiration CT-data and voxel-to-voxel mapping, which Solyanik described as “density mapping”. Among the three evaluated methods, density mapping showed the best correlation with the ratio of residual volume to total lung capacity (RV/TLC).

Parametric response mapping, similar to density mapping, involves assessing the attenuation of each voxel on inspiration and expiration after applying an elastic registration algorithm. Voxels with attenuation ≥950 HU and <−810 HU at inspiration and <−856 at expiration are considered to represent functional small airways disease (PRMfSAD). Belloli et al. [11] evaluating a retrospective cohort of 52 LTx patients reported that PRMfSAD values ≥ 30% were the strongest predictor of survival in a multivariable model including BOS grade and baseline FEV1% predicted. Verleden et al. [10] used PRM to monitor BOS progression in a retrospective study including 20 BOS and 20 controls (no restrictive CLAD phenotype). They observed an increase at time of BOS diagnosis. They also reported that patients who died from BOS had significantly higher PRMfSAD than living patients. Galban et al. investigated the use of PRM as an imaging biomarker in the diagnosis of BOS. They found that PRMfSAD > 28% was highly indicative of BOS occurrence, whether a concurrent infection was present or not [19]. 

Other authors have developed quantitative methods only requiring inspiratory CT data. This was the case for Horie et al. [18], who evaluated CT lung density histograms on a single inspiratory CT in CLAD patients after lung segmentation. Indeed, lung fibrosis associated with the RAS phenotype of CLAD is likely to increase lung density, whereas mosaic perfusion secondary to BOS has an invert effect, both being detectable when analyzing the lung density histogram: the right-sided tail reflects processes increasing lung density whereas the left-sided tail reflects processes decreasing lung density, such as mosaic attenuation in areas of BOS. Their objective was threefold, to evaluate prognosis after CLAD onset, distinguish between BOS and RAS phenotypes, and evaluate the prognosis within BOS and RAS patient groups separately. They evaluated the quantitative density metrics (QDM) defined as ratios of the right and left quantile weights of the histogram, reflecting the left vs. right asymmetry of the histogram. There was a statistically significant difference in QDMs between RAS and BOS patients. Higher QDM values were significantly associated with decreased survival. The authors concluded that this quantitative analysis of CT images was associated with survival after the onset of CLAD and was able to differentiate RAS and BOS phenotypes. QDM measurement has been used by the same authors [25] to predict the risk of subsequent CLAD in patients with CLAD-0p, defined as a drop in FEV1 to 81–90% of baseline. Higher QDM values were associated with a shorter time between CLAD-0p-CT and CLAD.

Fewer quantitative CT methods have been developed for the RAS phenotype of CLAD. Measurement of inspiratory and expiratory CT lung volumes can be easily performed and CT volumetry has been shown to differentiate RAS patients from BOS patients due to the strong positive correlation between inspiratory lung CT volumes and TLC [20]. Computed tomography is particularly useful for detecting CLAD affecting a single lung [7,21], because the unaffected contralateral lung compensates for the deficit in lung function on PFTs. A decrease over time in the difference between inspiratory and expiratory lung volumes has been reported for obstructive and restrictive CLAD phenotypes [21].

PRM is another method allowing quantitative evaluation of patients with the RAS phenotype of CLAD, who have persistent parenchymal opacities and pleural thickening. Voxels with attenuation values ≥−810 HU at inspiration represent parenchymal disease (PRMPD). In the study by Belloli et al. [11] evaluating 22 LTx patients, those with concurrent declines in both FEV1 and FVC (e.g., RAS phenotype) were found to have significantly more PRMPD than their control group, even after adjusting for age, baseline FEV1% predicted, and baseline FVC % predicted. The prognostic value of PRMPD was not mentioned and should probably be evaluated in a larger patient group. 

To be clinically useful, quantitative methods should not require multiple complex algorithms or manual steps to correct segmentation, which is the case with computer-assisted airway morphometry. In addition, these methods only assess the BOS phenotype of CLAD and have limited diagnostic value. Simpler methods based on CT volumetry are more accessible, with the assessment of lung volume and lobe attenuation on inspiration and expiration of interest for all CLAD phenotypes.

Regarding the processing of imaging data by artificial intelligence for CLAD diagnosis, the current literature is scarce. Classical machine learning methods such as the support vector machine (SVM) using quantitative CT metrics were of interest to diagnose BOS following unilateral LTx, as previously mentioned [16]. SVM was also used to evaluate multiple combinations of functional respiratory indexes (FRI) for BOS prediction at six months after transplantation [12]. A maximal accuracy of 85% was obtained by combining three baseline FRI features: the right middle lobe volume at total lung capacity (inspiratory CT scan), the right upper lobe airway resistance and the central airway surface at functional residual capacity (expiratory CT). Deep learning represents a major advance in the field of artificial intelligence applied to medical imaging, but requires a large amount of data [26,27]. As previously highlighted, most series on quantitative imaging for CLAD evaluation post LTx are based on a limited number of patients, because lung transplantation is not a common procedure. In 2015, only 14 centers reported performing 50 or more LTx per year [28]. Therefore, the standardization of imaging follow-up and data sharing through a common registry is crucial before considering the development of deep learning algorithms for the prediction and early diagnosis of CLAD.

## 4. Conclusions

Quantitative imaging methods offer the opportunity to perform reader-independent assessment of the airways and lung parenchyma in LTx patients. Although said to be automatic, most methods still require significant time-consuming manual corrections, and require the availability of segmentation and elastic registration algorithms, which limits their direct use in clinical routine. However, the results obtained in the studies published to date demonstrate the prognostic impact of methods such as parametric response mapping quantifying functional small airway disease (PRMfSAD) or quantitative density metrics (QDM). The performance for the prediction or early detection of CLAD needs to be strengthened, which could be an objective of deep learning-based methods. Developing prediction models is indeed important to improve outcomes for patients who are developing CLAD and better understand the underlining pathophysiology.

## Figures and Tables

**Table 1:** Definition of different CLAD phenotypes.

Phenotypes	Physiological Changes	Radiological Changes
**CLAD** (Chronic lung allograft dysfunction)	Persistent ≥ 20% decline in FEV1 (on the basis of 2 FEV1 values at least 3 weeks apart) compared with the baseline value, defined as the mean of the best 2 post-operative FEV1 measurement values, in the absence of other etiologies such as infection or acute rejection	
**BOS** (Bronchiolitis obliterans syndrome)	Persistent ≥ 20% decline in FEV1 compared with the baseline value (=CLAD definition) AND obstructive ventilatory defect (FEV1/forced vital capacity [FVC] < 0.7)	
**RAS** (Restrictive allograft syndrome)	Persistent ≥ 20% decline in FEV1 compared with the baseline value (=CLAD definition) AND ≥10% decline in TLC relative to baseline	Persistent opacities on chest imaging
**Mixed phenotype**	Persistent ≥ 20% decline in FEV1 compared with the baseline value (=CLAD definition) AND combination of obstructive and restrictive ventilatory defect (FEV1/FVC < 0.7 and a TLC ≤ 90% of baseline)	Persistent opacities on chest imaging
**Undefined phenotype (1)**	Persistent ≥ 20% decline in FEV1 compared with the baseline value (=CLAD definition) AND combination of obstructive and restrictive ventilatory defect (FEV1/FVC < 0.7 and a TLC ≤ 90% of baseline)	
**Undefined phenotype (2)**	Persistent ≥ 20% decline in FEV1 compared with the baseline value (=CLAD definition) AND obstructive ventilatory defect (FEV1/FVC < 0.7) and no decline in TLC	Persistent opacities on chest imaging

**Table 2:** Overview of CT quantification methods for CLAD.

Author Year	Year	Study Design/Number of Patients	Time of CT Evaluation	Software	Main Quantification Parameters	Main Results
**Airway measurement methods**						
Dettmer [9]	2014	Prospective study 141 patients (25 BOS+)	6, 12, 24 months after LTx	MeVis Airway Examiner	WT, WA%, WTdiff between inspiration and expiration on two selected bronchi B01 and B10	Greater WA% on inspiration in BOS+
Doellinger [8]	2016	Retrospective study 26 patients (12 BOS+)	All available CT scans after LTx	YACTA module v.1.0.7.16	∆WT and ∆WA%: temporal change of WT and WA%;	Temporal changes of WT and WA% showed significant differences between BOS+ and BOS−
Gazourian [14]	2017	Retrospective study 66 patients (20 controls, 22 BOS non progressors and 24 BOS progressors)	non-volumetric CT closest to baseline FEV_1_ and 2 follow-up CT scans	Airway Inspector (www.airwayinspector.org)	Internal lumen perimeter Lumen airway Airway vessel (A/V) ratio	Increase in the A/V ratio on follow-up CT scans for BOS progressors
Barbosa [12]	2018	Retrospective study 71 patients (41 BOS+)	2 CT scans (>3 months apart)	Mimics, TGrid 14.0 and Fluent 14.0	Airway volumes Airway resistances	Increase in central airway volume on expiratory CT in BOS+ Smaller airway volumes and airway surfaces and higher airway resistances at baseline in BOS developers
**Vascular measurement method**						
Gazourian [14]	2017	Retrospective study 22 patients (13 BOS+)	2 volumetric CT angiographies after LTx	Bronchi: Airway Inspector Vessels: Upper thresholding (cut-off level of −500 HU) and use of a connected components technique	Vessel cross sectional area (CSA) Airway lumen area Airway/Vascular ratio (A/V ratio)	Overtime decrease in CSA in BOS+ Overtime Increase in A/V ratio in BOS+
**Parenchyma-based methods: quantification of air trapping**						
Belloli [11]	2017	Retrospective study (22 BOS+ and controls matched by time from LTx; 52 BOS+)	Date of BOS	Lung segmentation: In-house algorithm Insp/Expiratory registration algorithm: Elastix	Parametric response mapping (PRM): Density-based quantification of air trapping (PRM^fSAD^) and parenchymal disease (PRM^PD^)	FEV_1_ decline associated with higher PRM^fSAD^ FEV_1_ and FVC decline associated with higher PRM^PD^ PRM^fSAD^ ≥ 30% strongest predictor of death
Verleden [10]	2016	Retrospective study 40 patients (20 BOS+)	CT scans before, at the time of and after of the diagnostic of BOS	Lung segmentation: In-house algorithm Insp/Expiratory registration algorithm: Elastix	Density-based quantification of air trapping (PRM^fSAD^), parenchymal disease (PRM^PD^) and normal lung (PRM^Normal^)	Increase in PRM^fSAD^ and decrease in PRM^Normal^ in BOS+ No difference in PRM^fSAD^ between BOS- and BOS+ before the diagnosis of BOS
Solyanik [15]	2015	Prospective study 147 patients (34 with air-trapping)	CT at 6 months after LTx	Not mentioned	Density-based quantification of air trapping (EXP_-790 HU to -950 HU_, E/I-MLD) Density mapping: voxel-to-voxel insp/expiration mapping	DM has the highest correlation to RV/TLC (r = 0.663, *p* < 0.001) DM and E/I-ratio MLD showed better correlation with RV/TLC than EXP_-790HU to -950HU_
Barbosa [16]	2017	Retrospective study 174 patients (98 BOS+)	CTs within 9 years after LTx	ANTs package	- Lung volume in inspiration and expiration - Lung volume difference between insp and expiration - Density-based quantification of air trapping (EXP<_-856 HU_, voxel volume with <75 HU increase on expiration)	Only 59% of qCT parameters associated with BOS+ BOS prediction model combining qCT and PFT parameters outperforms model based on PFTs alone in the unilateral LTx group
Dettmer [17]	2018	Prospective study 51 patients (17 BOS+)	Last CT within 1 year before BOS diagnostic First CT within 1 year after BOS diagnostic	Mevis Pulmo	Density-based quantification of air trapping (E/I-MLD ratio, E/I Volumes, density percentiles)	Significant increase in E/I-Volumes and decrease in E/I-MLD in BOS+ Changes more pronounced in the lower lobes Highest AUC for 10th percentile on expiration (0.903) and E/I-MLD ratio (AUC: 0.886)
Horie [18]	2018	Retrospective study 74 patients (23 RAS, 51 BOS)	CT performed ±4 months from CLAD and/or RAS/BOS onset.	Lung segmentation on Vitrea workstation	Lung volume and MLD on inspiration Quantitative density metrics (QDM) defined as ratios of the right and left quantile weights of the density histogram on inspiratory CT	Significant difference of Lung volume and MLD in BOS and RAS patients Hazard ratio for death 3.2 times higher at the 75th percentile of QDM1compared to the 25th percentile
Saito [20]	2016	Retrospective study 63 patients (19 RAS, 44 BOS)	CT performed at baseline and time of CLAD onset	Lung segmentation on Vitrea workstation	Lung volume on inspiration	Decrease in CT lung volume in RAS patients CT volumetry < 90% baseline had an accuracy of 0.937 for differentiating RAS from BOS
Saito [21]	2018	Retrospective study 58 patients, 14 CLAD	CT performed 3, 6, and 12 months after LTx and once yearly thereafter	Lung segmentation on Synapse Vincent workstation	Lung volume on inspiration and expiration Evaluation of Δlung volume over time (difference between inspiration and expiration)	Δlung volume onset/baseline significantly decreased in the CLAD group 0.80 cutoff had an AUC of 0.87

LTx: lung transplantation; WT^:^ wall thickness; WA%: wall area percentage = ratio bronchial wall/total area (bronchial wall+ bronchial lumen); WTdiff: difference of wall thickness between expiration and inspiration; A/V ratio: airway vessel ratio; ∆WT and ∆WA%: temporal changes of WT and WA%; PRM^fSAD^: Parametric Response Mapping representing functional small airways disease; PRM^PD^: Parametric Response Mapping representing parenchymal disease; PRM^Normal^: Parametric Response Mapping representing normal pulmonary parenchyma; EXP_-790 HU to -950 HU_: percentage of voxels with attenuation values from -790 HU to -950 HU on expiration; DM: density mapping; E/I-MLD: expiratory-to-inspiratory mean lung density ratio; E/I- Volumes: expiratory-to-inspiratory volume ratio; RV/TLC: residual volume/total lung capacity qCT: quantitative CT.

## Data Availability

Not applicable.

## References

[1] Chambers D.C., Yusen R.D., Cherikh W.S., Goldfarb S.B., Kucheryavaya A.Y., Khusch K., Levvey B.J., Lund L.H., Meiser B., Rossano J.W. (2017). The Registry of the International Society for Heart and Lung Transplantation: Thirty-Fourth Adult Lung and Heart-Lung Transplantation Report—2017; Focus Theme: Allograft Ischemic Time. J. Heart Lung. Transplant..

[2] Verleden G.M., Glanville A.R., Lease E.D., Fisher A.J., Calabrese F., Corris P.A., Ensor C.R., Gottlieb J., Hachem R.R., Lama V. (2019). Chronic Lung Allograft Dysfunction: Definition, Diagnostic Criteria, and Approaches to Treatment—A Consensus Report from the Pulmonary Council of the ISHLT. J. Heart Lung. Transplant..

[3] Hota P., Dass C., Kumaran M., Simpson S. (2018). High-Resolution CT Findings of Obstructive and Restrictive Phenotypes of Chronic Lung Allograft Dysfunction: More Than Just Bronchiolitis Obliterans Syndrome. Am. J. Roentgenol..

[4] DerHovanessian A., Todd J.L., Zhang A., Li N., Mayalall A., Finlen Copeland C.A., Shino M., Pavlisko E.N., Wallace W.D., Gregson A. (2016). Validation and Refinement of Chronic Lung Allograft Dysfunction Phenotypes in Bilateral and Single Lung Recipients. Ann. Am. Thorac. Soc..

[5] Levy L., Huszti E., Renaud-Picard B., Berra G., Kawashima M., Takahagi A., Fuchs E., Ghany R., Moshkelgosha S., Keshavjee S. (2020). Risk Assessment of Chronic Lung Allograft Dysfunction Phenotypes: Validation and Proposed Refinement of the 2019 International Society for Heart and Lung Transplantation Classification System. J. Heart Lung. Transplant..

[6] Suhling H., Dettmer S., Greer M., Fuehner T., Avsar M., Haverich A., Welte T., Gottlieb J. (2016). Phenotyping Chronic Lung Allograft Dysfunction Using Body Plethysmography and Computed Tomography. Am. J. Transplant..

[7] Philippot Q., Debray M.-P., Bun R., Frija-Masson J., Bunel V., Morer L., Roux A., Picard C., Jebrak G., Dauriat G. (2020). Use of CT-SCAN Score and Volume Measures to Early Identify Restrictive Allograft Syndrome in Single Lung Transplant Recipients. J. Heart Lung. Transplant..

[8] Doellinger F., Weinheimer O., Zwiener I., Mayer E., Buhl R., Fahlenkamp U.L., Dueber C., Achenbach T. (2016). Differences of Airway Dimensions between Patients with and without Bronchiolitis Obliterans Syndrome after Lung Transplantation-Computer-Assisted Quantification of Computed Tomography. Eur. J. Radiol..

[9] Dettmer S., Peters L., de Wall C., Schaefer-Prokop C., Schmidt M., Warnecke G., Gottlieb J., Wacker F., Shin H. (2014). Bronchial Wall Measurements in Patients after Lung Transplantation: Evaluation of the Diagnostic Value for the Diagnosis of Bronchiolitis Obliterans Syndrome. PLoS ONE.

[10] Verleden S.E., Vos R., Vandermeulen E., Ruttens D., Bellon H., Heigl T., Van Raemdonck D.E., Verleden G.M., Lama V., Ross B.D. (2016). Parametric Response Mapping of Bronchiolitis Obliterans Syndrome Progression after Lung Transplantation. Am. J. Transplant..

[11] Belloli E.A., Degtiar I., Wang X., Yanik G.A., Stuckey L.J., Verleden S.E., Kazerooni E.A., Ross B.D., Murray S., Galbán C.J. (2017). Parametric Response Mapping as an Imaging Biomarker in Lung Transplant Recipients. Am. J. Respir. Crit. Care Med..

[12] Barbosa E.J.M., Lanclus M., Vos W., Van Holsbeke C., De Backer W., De Backer J., Lee J. (2018). Machine Learning Algorithms Utilizing Quantitative CT Features May Predict Eventual Onset of Bronchiolitis Obliterans Syndrome After Lung Transplantation. Acad. Radiol..

[13] Tissot A., Danger R., Claustre J., Magnan A., Brouard S. (2019). Early Identification of Chronic Lung Allograft Dysfunction: The Need of Biomarkers. Front. Immunol..

[14] Gazourian L., Ash S., Meserve E.E.K., Diaz A., Estepar R.S.J., El-Chemaly S.Y., Rosas I.O., Divo M., Fuhlbrigge A.L., Camp P.C. (2017). Quantitative Computed Tomography Assessment of Bronchiolitis Obliterans Syndrome after Lung Transplantation. Clin. Transplant..

[15] Solyanik O., Hollmann P., Dettmer S., Kaireit T., Schaefer-Prokop C., Wacker F., Vogel-Claussen J., Shin H. (2015). Quantification of Pathologic Air Trapping in Lung Transplant Patients Using CT Density Mapping: Comparison with Other CT Air Trapping Measures. PLoS ONE.

[16] Barbosa E.M., Simpson S., Lee J.C., Tustison N., Gee J., Shou H. (2017). Multivariate Modeling Using Quantitative CT Metrics May Improve Accuracy of Diagnosis of Bronchiolitis Obliterans Syndrome after Lung Transplantation. Comput. Biol. Med..

[17] Dettmer S., Suhling H., Klingenberg I., Otten O., Kaireit T., Fuge J., Kuhnigk J.M., Gottlieb J., Haverich A., Welte T. (2018). Lobe-Wise Assessment of Lung Volume and Density Distribution in Lung Transplant Patients and Value for Early Detection of Bronchiolitis Obliterans Syndrome. Eur. J. Radiol..

[18] Horie M., Salazar P., Saito T., Binnie M., Brock K., Yasufuku K., Azad S., Keshavjee S., Martinu T., Paul N. (2018). Quantitative Chest CT for Subtyping Chronic Lung Allograft Dysfunction and Its Association with Survival. Clin. Transplant..

[19] Galbán C.J., Boes J.L., Bule M., Kitko C.L., Couriel D.R., Johnson T.D., Lama V., Telenga E.D., van den Berge M., Rehemtulla A. (2014). Parametric Response Mapping as an Indicator of Bronchiolitis Obliterans Syndrome after Hematopoietic Stem Cell Transplantation. Biol. Blood Marrow Transplant..

[20] Saito T., Horie M., Sato M., Nakajima D., Shoushtarizadeh H., Binnie M., Azad S., Hwang D.M., Machuca T.N., Waddell T.K. (2016). Low-Dose Computed Tomography Volumetry for Subtyping Chronic Lung Allograft Dysfunction. J. Heart Lung. Transplant..

[21] Saito M., Chen-Yoshikawa T.F., Nakamoto Y., Kayawake H., Tokuno J., Ueda S., Yamagishi H., Gochi F., Okabe R., Takahagi A. (2018). Unilateral Chronic Lung Allograft Dysfunction Assessed by Biphasic Computed Tomographic Volumetry in Bilateral Living-Donor Lobar Lung Transplantation. Transplant. Direct.

[22] Weinheimer O., Achenbach T., Bletz C., Duber C., Kauczor H.U., Heussel C.P. (2008). About Objective 3-d Analysis of Airway Geometry in Computerized Tomography. IEEE Trans. Med. Imaging.

[23] Achenbach T., Weinheimer O., Biedermann A., Schmitt S., Freudenstein D., Goutham E., Kunz R.P., Buhl R., Dueber C., Heussel C.P. (2008). MDCT Assessment of Airway Wall Thickness in COPD Patients Using a New Method: Correlations with Pulmonary Function Tests. Eur. Radiol..

[24] Achenbach T., Weinheimer O., Buschsieweke C., Heussel C.P., Thelen M., Kauczor H.U. (2004). Fully automatic detection and quantification of emphysema on thin section MD-CT of the chest by a new and dedicated software. Rofo.

[25] Horie M., Levy L., Houbois C., Salazar P., Saito T., Pakkal M., O’Brien C., Sajja S., Brock K., Yasufuku K. (2019). Lung Density Analysis Using Quantitative Chest CT for Early Prediction of Chronic Lung Allograft Dysfunction. Transplantation.

[26] Chassagnon G., Vakalopoulou M., Paragios N., Revel M.-P. (2020). Artificial Intelligence Applications for Thoracic Imaging. Eur. J. Radiol..

[27] Chassagnon G., Vakalopolou M., Paragios N., Revel M.-P. (2020). Deep Learning: Definition and Perspectives for Thoracic Imaging. Eur. Radiol..

[28] Chambers D.C., Cherikh W.S., Harhay M.O., Hayes D., Hsich E., Khush K.K., Meiser B., Potena L., Rossano J.W., Toll A.E. (2019). The International Thoracic Organ Transplant Registry of the International Society for Heart and Lung Transplantation: Thirty-Sixth Adult Lung and Heart-Lung Transplantation Report-2019; Focus Theme: Donor and Recipient Size Match. J. Heart Lung. Transplant..

